# Community engagement to develop a dialogue-drama on adolescent pregnancy in a marginalised migrant population on the Thailand-Myanmar border: an ethnographic approach to participatory action research

**DOI:** 10.1080/16549716.2024.2328893

**Published:** 2024-11-08

**Authors:** Saw San Soe, Saw Thwe Paw, Mu Lwel Tha Dah, Day Mu Dah, Thae Thae Naing, K Mwee Hser, Solomon Naw, Htet Khaing Lu, Kanjana Winyoorat, Primprapaporn Thongdee, Saw Win Tun, Poe Poe, Rose McGready, Poe Christ, Htee K Poung, Win Win Cho, Hser Nay Wah, Htoo Hser, Saw Phee Do, Paw Bway Shee, Bulakorn Tinoi, Prapatsorn Misa, May Myo Thwin, Ladda Kajeechiwa

**Affiliations:** Shoklo Malaria Research Unit, Mahidol-Oxford Tropical Medicine Research Unit, Faculty of Tropical Medicine, Mahidol University, Mae Sot, Thailand

**Keywords:** Participatory action research, Karen, Burmese, pregnancy, adolescence

## Abstract

**Background:**

Communities in which adolescent pregnancy and safe abortion care are taboo may benefit from culturally appropriate information, education, and communication.

**Objective:**

This ethnographic and participatory action research (PAR) elicited community members’ perceptions to adolescent pregnancy: which then informed dialogue-drama development in Burmese and Karen language for undocumented migrants on the Thailand-Myanmar border.

**Methods:**

PAR was conducted in Karen and Burmese language. Interviews and discussions elicited perceptions of community members about adolescent pregnancy. These were analysed for themes and using the fishbone technique, to determine the objectives for the drama. After developing the structure and content of the drama it was piloted, revised, and performed in communities. Responses and impact of the drama were recorded. The team reflected on the drama as a method for health messaging.

**Results:**

In 2022, themes of responsibility, communication, and experiences of adolescent pregnancy emerged from 10 interviews and 6 discussions with community members. The fishbone technique established three dramatic objectives, woven into a teenage love story with an unplanned pregnancy, to raise community awareness of i) adolescent pregnancy, ii) contraception, and iii) choice in unexpected pregnancy. Post-drama feedback from 11 migrant communities (1,238 participants) was positive although some community members voiced concerns. Given the logistical challenges of conducting the drama in person, a film will be created for wider dissemination.

**Conclusions:**

Participatory action research resulted in a culturally-nuanced performance, with communities requesting further performances and awareness on adolescent pregnancy and safe abortion care. Video is likely to be a more sustainable option.

## Paper context

- **Main findings:** A participatory-action approach involving community members and adolescents resulted in a dialogue-drama which raised awareness of adolescent pregnancy, contraception, and choice in the event of an unplanned pregnancy.
- **Added knowledge:** The dialogue-drama was a useful tool, augmenting discussion of traditionally taboo issues including adolescent pregnancy and unsafe abortion.
- **Global health impact for policy and action:** The community driven dialogue-drama was well received and recommended by the community to address other health issues and hence this ethnographic method is potentially useful for similar global health settings.

## Background

While education that promotes contraception is the most effective intervention to reduce unintended pregnancy [1], if pregnancy does occur, supporting health-promoting choices is a key component of care particularly for adolescents, their families and communities. Nevertheless, teenage sex, pregnancy, and safe abortion care remain highly stigmatised topics [2]. Understanding community sensitivities and perceived need is critical before investing in information, education and communication (IEC) materials directed at adolescents whose care remains predominantly a parental responsibility [1].

The performing arts are one avenue to develop IEC materials to address social issues in healthcare as it can foster culturally appropriate empathy and communication [3]. Evidence suggests that stories as a method of communication can influence beliefs and behaviours [4]. Stories that are relatable to the community are particularly influential for youth. Cognitive and affective engagement can stimulate behaviour changes [5] important in promoting healthy choices for sexual and reproductive health (SRH) among adolescents early on in life. In Myanmar, traditional performing arts troupes have retained popularity as they entertain and engage through storytelling, dancing, and singing [6]. The troupes relate the struggles and resilience of the people despite both censorship and appropriation of ‘Burmese traditions’ by the military regime [7].

The World Health Organization [8] defines adolescence as between 10 and 20 years of age [9]. In a study of migrants from Myanmar residing on the Thailand-Myanmar border conducted from 1998 to 2017, adolescents made up approximately one in six of all pregnancies [10]. Qualitative work from the same setting with adolescent pregnant women and adolescent husbands and boys reported: ‘the main underlying reasons for adolescent pregnancy were associated with traditional views and stigma on SRH issues, resulting in a knowledge gap on contraception and life skills necessary to negotiate sexual and reproductive choices, in particular for unmarried adolescents’ [11]. The risk of adolescent pregnancy, unsafe abortion, and/or suicide due to an unplanned pregnancy can be reduced by [12–16]: accessible adolescent friendly reproductive health services [17], healthy parent-adolescent communication [18], positive societal attitudes to gender equity [19] and sexual health education in schools [20].

The objective of this ethnographic and participatory action research (PAR) was to understand community members’ perceptions about adolescent pregnancy and utilise this information to develop a dialogue-drama (referred to hence onwards as drama). This manuscript describes the community-based PAR used to develop and assess a drama as a method of health messaging for challenging issues in low-resource communities.

## Methods

### Qualitative approach

**Figure 1.**
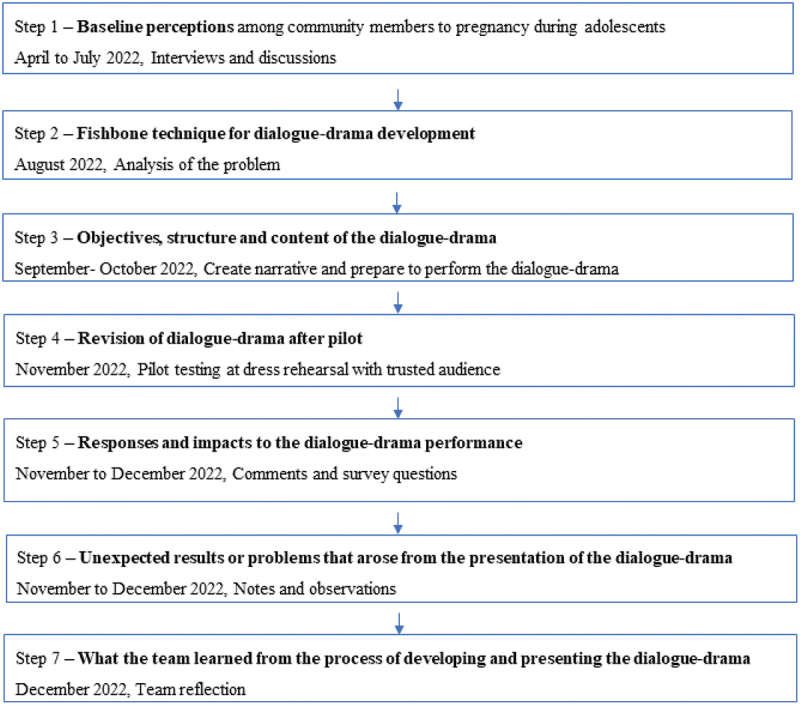
Study flow chart.

PAR is a method that permits the community to drive the research as those most affected are involved in inquiry, design, and in executing the process, which is essential for creating social change [21]. PAR was used as an ethnographic approach in the undocumented migrant communities to understand perceptions of adolescent pregnancy. Stigma and misconceptions were already recognised as problematic from earlier published work in the area [11,22,23], and attitudes and emotions may change over time. Social justice for adolescent pregnancy in undocumented migrants was a transformative paradigm that also provided a good fit for this activity [24,25]. The team planned the activity in sequential steps as outlined in the study flow (Figure 1).

### Community engagement team characteristics and reflexivity

The community engagement team of the Shoklo Malaria Research Unit – Borderland Health Foundation (SMRU-BHF) has been working for decades on both sides of the Thailand-Myanmar border on health including malaria, tuberculosis, and COVID-19. They have established trust within the community and work to improve health literacy for villagers on different projects, such as malaria elimination [26] through the use of traditional storytelling and picture-based IEC materials. The team is experienced in conducting surveys, interviews, and focus group discussions (FGD). All members of the team speak at least two languages, mostly Karen (two dialects Sgaw and Poe) and Burmese, with most also having experience with English and some with Thai language. Experienced team members (>40 years) guide and support younger less experienced members. This project included 31 members of the community engagement team comprising 20 women and 11 men. There were 5, 21 and 5 aged 18–24, 25–40, or >40 years, respectively. Most of the team members had education up to 16 years of age. The circumstances on the border limit further educational opportunities and while only five members of the team have had the opportunity to complete a tertiary qualification, these characteristics of the Team enhanced dialogue related to concerns of migrants and villagers.

### Context

Thailand is a regional hub within South-East Asia for migrant workers from Cambodia, Lao PDR, and Myanmar. Thailand is a country of origin, transit, and destination for documented and undocumented migrants, displaced persons (Thailand has not ratified 1951 UN Refugee Convention), and asylum seekers. The UN reports intensification of migration to Thailand from an estimated 3.7 million in 2014 to 4.9 million in 2019 [27].

In Tak Province, Thailand, agriculture is largely supported by a workforce of undocumented Myanmar migrants who frequently live and work as family units. SRH services in Karen and Burmese are limited. While a strong system of public health in Thailand and access to universal healthcare well established, undocumented migrants are required to pay fees for services. This is unaffordable for low-paid migrant workers. The SMRU-BHF operates free SRH and Maternal, Newborn and Child Health (MNCH) services to migrant communities on both sides of the Thailand-Myanmar border in rural areas in Mae Ramat and Phop Phra districts of Tak Province. Migrant workers have frequently cut their schooling short [11] and health literacy is limited [28] and, of more than 70,000 registered pregnancies at SMRU-BHF, the proportion of adolescent pregnancy has remained high for two decades [10]. Unsafe abortion practices have also been documented in the border area [29,30]. Changes by the Constitutional Court of Thailand amended the legislature in early 2021 allowing abortion in cases of unwanted pregnancy [31].

### Baseline perceptions among community members to pregnancy during adolescents

To learn about community perceptions to adolescent pregnancy, the team met with migrants from Mae Ramat and Phop Phra districts in Tak Province. After receiving approval from the village headman, a designated meeting place, date, and time in the village were agreed upon and community volunteer health workers, teachers, and villagers were invited to attend. These were scheduled on the same day as antenatal care clinics. Adolescent pregnant women were approached in their own language if they were already attending clinic. A counselor is routinely present to provide support as part of the outreach. Four community engagement members aimed to complete 10 in-depth interviews (IDI) with individuals and conduct 6 focus discussion groups (FGD).

Before any interview, discussion, or video, verbal consent was obtained in the preferred language of the person, with no personal identifying data collected. The question guide for interviews and discussions is outlined in the Appendix file, and data was collected using notes taken during the interviews/discussions.

### Fishbone technique and drama development

The community engagement team had received prior training on the Fishbone technique [32,33], which they used to develop the drama given the problem of adolescent pregnancy. Eighteen team members worked over a four-month period reviewing transcripts of the notes of interviews and discussion. Working in three groups, each identified the most important findings. The groups shared their findings and the items were combined or discarded in an iterative process until there was consensus of the most important problems (the fish tail). Bite size or short sentences of the transcripts were photocopied and the group separated them into effects (reported experience) and causes (fish skeleton). From this, the desire for the drama emerged (fish head). The effects and causes were written into the script to ensure realism and sentiments from border communities were embedded in the narrative. In the drama, the story rises to a climax and is then paused for a dialogue. The main actors and a host interact with the audience for the ‘Dream Scene’: a different ending to the climax they just saw. The Dream Scene is then acted with the ideas of the drama team to reflect on how the issues could be alternatively resolved.

### Drama feedback

Drama feedback was collected by community members immediately with the audience and after the audience went home. Immediately after the drama, there was the dialogue conducted by host between all the characters and the audience in order to respond to questions and comments from the audience. Three community-engagement team members were notetakers and the video camera man was also present to record responses. There were three quick questions asking for a show of hands including:

- Did you like the drama performance you just saw?
- Do you think you have increased knowledge on adolescent pregnancy after seeing this drama performance?
- In other health-related topics, do you think drama can be used as a tool to increase understanding of health knowledge?

This was followed by three open-ended questions that audience members responded to. These questions included:

- What are your main takeaway messages after watching this drama show?
- Who else would you recommend to watch this drama show and why?
- We value your feedback and for us to improve in the future, do you have any suggestions about our drama performance?

Community members available after the drama were requested to respond to a survey of nine questions:

1. How did you feel about this drama show?
2. What messages did you get from the drama show?
3. Are there any parts or any scenes related to your experiences?
4. What do you think about the use of the Dream Scene and do you agree/disagree with it and why?
5. Did you find any scene sensitive and why and how would you like to change it? In your community, when there is a need to discuss about difficult topic/subject, how do you do it, and do you have suggestions for us?
6. Do you think this drama show can increase understanding and knowledge about adolescent pregnancy in your community?
7. Do you think this drama show can create/generate a friendly environment where people feel free to talk or discuss about adolescent pregnancy in your community?
8. With different topics, what is your view on using drama to deliver health message in the future?
9. Who else should see this drama show and why?

### Internal review

The team conducted an internal review after the data analysis and drama were completed to reflect on future approaches for IEC tools in migrant border communities. Discussion points included: Were the objectives of the drama met, why or why not? What should be the next step? How can we improve? The team divided into groups and each wrote responses and reported answers back to the group.

### Data security

Notes created during community engagement were kept in locked project filing cabinet when not in use. Video material was kept in a password-protected drive only available to three media team members.

### Data analysis

All information from interviews and discussions was collected in Karen and Burmese language. The community engagement team was responsible for all aspects of transcription and translation.

Transcripts and notes of interviews and discussions on perceptions were analysed with the use of content analysis. In the first stage, codes were developed by assigning names to small sections of the interview transcripts for both Burmese and Karen responses. Next, the most salient codes were identified and developed into themes that captured the most important issues. Codes that had the same meaning in both languages were grouped into a single code. All codes were present in both languages. No programme was used for qualitative analysis to allow the transcript analysis to take place in local languages. The fishbone technique was in Burmese and later transcribed to English for sharing. There was no a priori plan for analysis of community member drama comments and the team decided to use the same themes that emerged from the baseline study of perceptions.

The team were motivated to produce a drama that was meaningful to the community that would engage and empower them. There was no benefit to the team to take short cuts when discussing in the community or in falsifying statements.

## Results

This participatory action project was conducted from April to December 2022 (Figure 1).

### Baseline perceptions among community members to pregnancy during adolescents

There were 10 interviews, three with pregnant adolescents, three with migrant village health volunteers, three with villagers and one with a school teacher, of whom one was Karen and nine were Burmese (7 women, 3 men). There were six discussions with three conducted in Karen and three in Burmese. Two discussions were conducted with health workers providing ANC and family planning clinics in the community (*n* = 8, *n* = 7; 10 women, 5 men); and four with community members who included villagers, teachers, and community village health volunteers (*n* = 16, *n* = 10, *n* = 16, *n* = 16; 33 women, 25 men).

Three consistent themes emerged regarding community perceptions: responsibility, adolescent communication, and experiences of adolescent pregnancy (Table 1). Responsibility of adolescent pregnancy was directed more towards the mother than the father, however, the wider community or village was also perceived to be involved in the care of adolescents. Communication from an adolescent perspective was perceived negatively as parental pressure, scolding, and criticism. This was contrary to community members’ perceptions which were open to adolescent relationships as a natural and normal process and part of growing up. Community members communicated a willingness to support adolescents and viewed learning and teaching about adolescent pregnancy in their communities and schools positively. Despite this openness, they perceived youth as being difficult to manage suggesting barriers to communication. There was precise communication regarding messaging:

Don’t get married early, Get married only after graduation,

**Table 1.** Baseline perceptions among community members about adolescent pregnancy from interviews and discussions.

Themes	Perceptions	Interviews	Discussions
Responsibility	Parental pressure	Adolescents felt parental pressure – the parents preferred a certain girl or boy to marry, not marriage for love or by choice.	Adolescent pregnancy is the “parents’ responsibility (especially the mother) to look after adolescents but the whole of society (migrant community) also had a responsibility to adolescents”.
	Village health volunteers	They (adolescents) often had a positive pregnancy test. Some adolescent marriage in the community was going well but others had fighting, divorce, induced abortion and suicide.	Adolescent pregnancy was not perceived as a problem if the couple were stable economically (employment) and by “status” (documentation).
Communication issues	Feelings	Adolescents felt there was gossip about them; they were scolded, criticized and experienced a sense of discrimination.	Community members held positive views about being consulted, they wanted to be consulted, they said they would be supportive with advice, encouragement and physical support. Some parents, especially mothers, said that they had talked about pregnancy to adolescents and a few teachers, health volunteers, and village leaders also said they had communicated on these topics.
	Support	Parents and relatives could be supportive with information during pregnancy, helping with food, shelter and safety. Some adolescents did not have family present for support.	Participants were comfortable while answering questions about love, sex and relationships. They are used to this happening naturally. They expressed that they have open views in their minds: it was natural for adolescents to try things out e.g. sex, but they (adolescents) were not always practical about dealing with problems.
	Message for other adolescents	Don’t get married early; get married only after graduation. Better to be sure that you are ready and able to arrange for all the things a family needs i.e. food, shelter, safety and medical cover.	The majority of participants felt that adolescents did not discuss or communicate with anyone about love, sex or relationships – it wasn’t talked about. Youth are difficult to manage.
Experiences of pregnancy	Confusion	Confusion over common symptoms of pregnancy, absent and late menstruation and what to do about it. Contraceptive information often came from the market and sometimes from parents and relatives, and was all different.	Ideas for supporting adolescents included: explore sexual reproductive education before getting married; learning *[about sexual and reproductive health]* should be fulfilled at a young age in order to avoid early pregnancy; information on use of family planning should be part of what students learn.
	Future	Extreme happiness, pleasant feelings and excitement; to later feelings of depression as it was not clear how to support a child in the current economic situation, worries about actually giving birth, and about the child’s future; to some not feeling overly or underly concerned (adolescent pregnancy is part of the norm).	Participants wanted adolescents to take time to know one another well before having sexual relationships.

and a pragmatic comment:

Better to be sure that you are ready and able to arrange for all the things a family needs i.e. food, shelter, safety and medical cover.

Experiences of adolescent pregnancy included confusion about basic sexual health including menstruation and contraception. Feelings of doubt, happiness, excitement, and fear surrounding the pregnancy and future were expressed, while community members wanted adolescents to create healthy relationships before sexual intercourse.

### Fishbone technique and drama development

**Figure 2.**
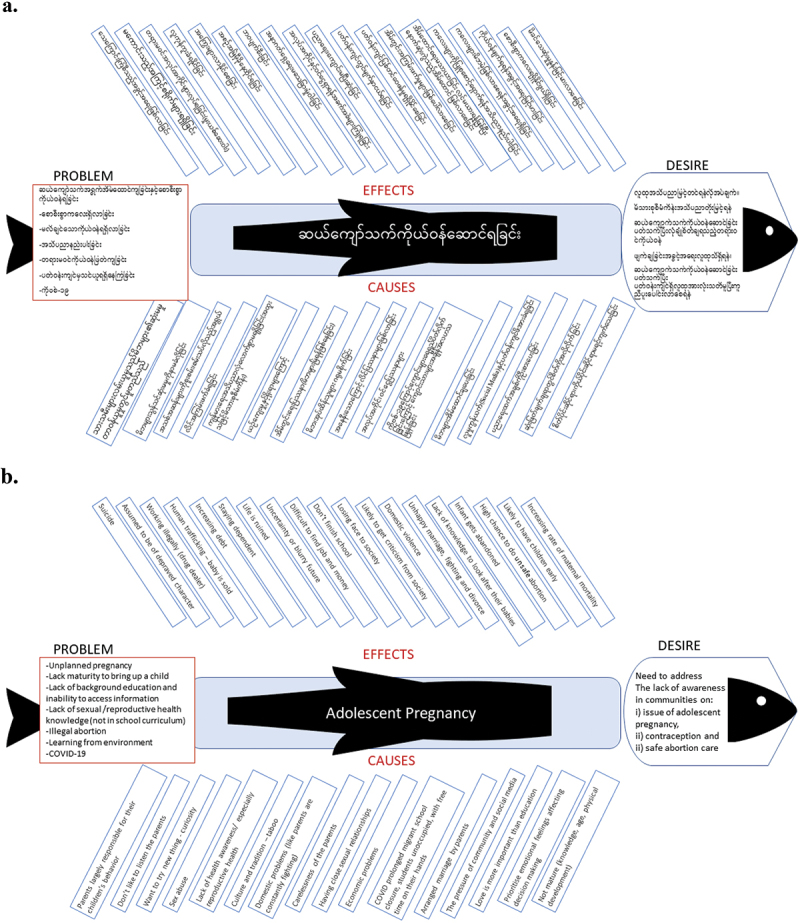
Fishbone in (a) Burmese and (b) English.

The Fishbone technique summarised the story of adolescent pregnancy informed by the community (Figure 2). The problems of adolescent pregnancy (Fish tail) included: unplanned pregnancy; limited education due to the border situation and with reduced literacy limiting access to information; lack of sexual/reproductive health knowledge (no comprehensive school curriculum); illegal abortion; youth learning from their environment; and being too immature to care for a child. The effects (experiences) of adolescent pregnancy expressed by community members were numerous, serious, and predominantly negative e.g. unsafe abortion, suicide, and not finishing school. The causes of adolescent pregnancy were attributed to: normal adolescent behaviour such as wanting to try new things while lacking maturity in decision-making; the negative influence of social media challenging taboos imposed by culture and tradition; and the importance of parental responsibility. This led to the desire (fish head) for the drama and these became the objectives of the drama.

### The objectives, structure, and content of the drama

The objective of the drama was to raise community awareness of adolescent pregnancy, contraception, and choice in the event of an unplanned pregnancy. The drama story revolves around an adolescent love story among migrant school students residing along the Thailand-Myanmar border. There are four main characters: Thae Su, her mother, Na Di, and Min Khant. The plot centres on Thae Su, a trusting 15 year-old girl who falls in love with a charming and popular classmate, Min Khant. Na Di, Thae Su’s best friend disapproves as Min Khant has the reputation of a playboy. The widowed mother of Thae Su is consumed by work and tries her best to provide for her family as a daily wage earner. Her hours are long and her conversations with her daughter superficial. Thae Su and Min Khant meet frequently and in private down at the riverside and Thae Su easily brushes off her mother’s queries about why she is home late. Thae Su and Min Khant do not know about contraception, and a brief shadow scene (frequently used in puppet shows in South-East Asia [34]) suggests they get sexually involved, resulting in an unplanned pregnancy. When Thae Su reveals the situation to Min Khant, he vehemently denies his role. The inconsolable Thae Su turns back to Na Di, confides in her, and asks for her help. Na Di’s advice is whispered in Thae Su’s ear. This is a symbolic moment as the audience hears nothing yet still know what she said – aligning with the taboo subject of unplanned pregnancy. It does not go well. As Thae Su does not ask for her help, her mother is unaware of the difficulties her daughter is facing until it is too late. Na Di knows things are not good when she sees so much blood on Thae Su’s dress. The mother does not know what has happened, and she is so distraught that her only daughter may be lost. The scene closes with Na Di in panic and the mother sobbing in despair.

The drama is paused at this critical moment for dialogue. The main characters interact with the audience asking questions and responding as they raise key points: What do you see as the problem? Each character has a problem – can you say what it is? How do you want the character to solve this problem? Apart from the characters, who else is responsible for this problem? The host encourages the audience to ‘dream’ about how they want the characters to change and what they can do differently. The drama is re-enacted as the Dream Scene, bringing in elements of the ‘changes’ suggested in the dialogue. In this section of the PAR, the values of audience are prioritised and their experiential knowledge is used to recognise problems and inequality and to envision alternatives.

Thae Su finally decides to confide in her mother. The scene turns from one of anger, shouting, crying, and disbelief, to absolute, unconditional love as her mother says they will go together to the health centre. At the health centre the health worker listens to the story of Thae Su and her mother. The health worker counsels and asks them to think about the three choices. First continue the pregnancy and think about who will take care of the baby, second continue the pregnancy and give the baby up for adoption and third, safe abortion care. The health worker suggests they do not have to decide immediately and to go home and think about their decision and return in a few days. The health worker reassures Thae Su and her mother that the staff will support their decision. On return, Thae Su and her mother and Min Khant say they will keep the pregnancy, and Min Khant will get a job and take care of his family.

The themes of the drama include teenage love, a mother's love, communication, and responsibility which are woven through the drama and in the dialogue as the audience is encouraged to realise how the actions of the characters have significant consequences. Tension is raised in the different scenes including Min Khant’s denial, the whisper of Na Di into Thae Su’s ear, Na Di seeing blood on the dress of her friend, the mother not realising what is going on although the audience does and starting on the walk to the health centre. Great tension is released with the kind reception of Thae Su and her mother, and clarity of information provided at the clinic by the health worker.

### How the team revised the drama after pilot testing

The pilot testing was a dress rehearsal performance in a relaxed setting in the Mae Sot area, with the audience including members of a Young Person’s Advisory Group (Y-PAG) and SMRU-BHF staff. This audience provided feedback. The host and actors faced their first round of live audience responses.

Following this performance, the characters and host learned they may not be able to answer all questions directly. With the notetakers having taken questions each was checked and when necessary the correct medical information was clarified. The team also decided to promote the same response for rude questions or comments along the lines of ‘that is not the language we are using in this show, can you rephrase?’

### Responses and impacts to the drama performance

**Figure 3.**
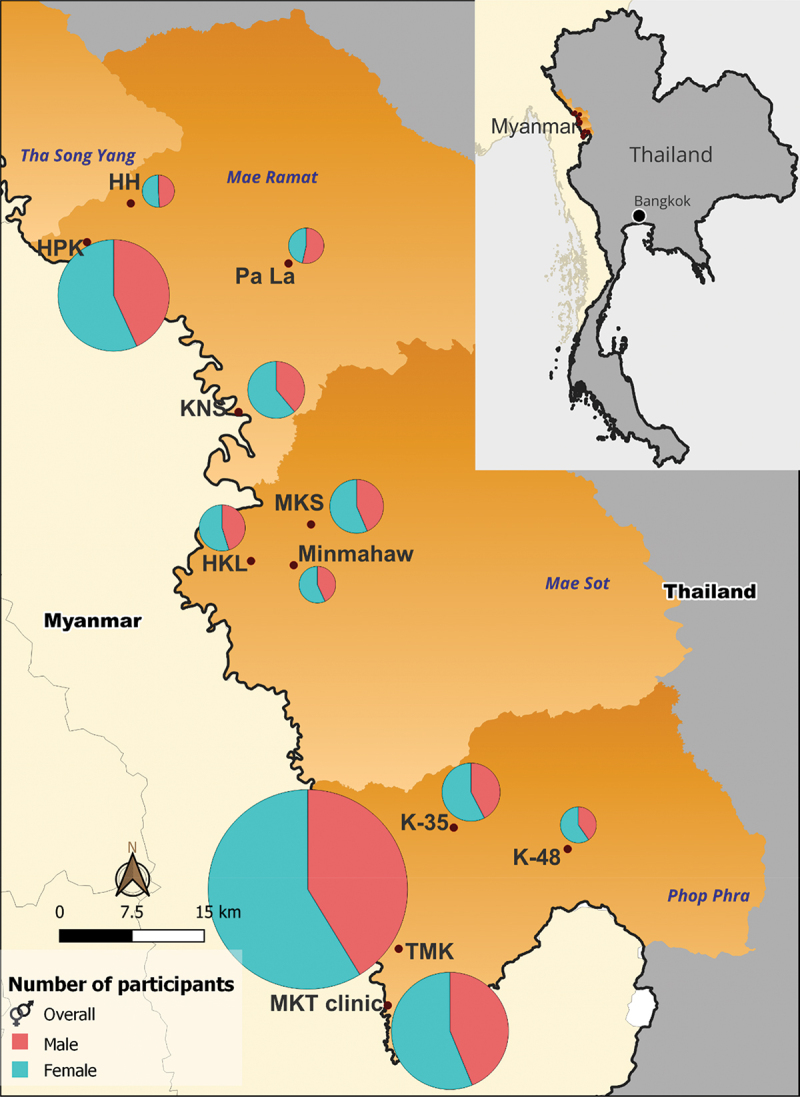
Sites of the drama performance in Tak Province, Thailand with proportional representation of participants by site and disaggregated by sex.

Migrant communities and schools from the two districts were approached for their interest in having the drama performed in their area. Of 13 that were approached two (15.4%) refused: one thought the topic was sensitive, while the other asked for something in return which was beyond the capacity of the community engagement team to provide. Of these 11 sites, eight were migrant or Thai-Karen communities and three were migrant schools in Mae Sot, Mae Ramat, and Prop Phra districts. Of a total audience of 1,238 the average [min-max] proportion of males and females less than 18 years of age per drama performance was 44.4% (237/534) [0–100%] and 43.5% (306/704) [0–100], respectively (Figure 3).

Immediate responses post performance by show of hands were positive; with nearly all the audience agreeing they liked it, that it increased their knowledge about adolescent pregnancy and would recommend others to see the drama. Comments were grouped by the same themes established in the baseline perceptions. In terms of responsibilities, the audience stressed relationships and care for adolescents:

A mother needs to have a close relationship with her daughter.

Parents have to make a close relationship with their children and consciously look after them.

Teachers should create comfortable and close relationships with their students. They have obligations of guiding them as they are considered as second parents.

There were frank comments about communication and finding the right person when considering unwanted pregnancy:

If there is any unwanted pregnancy, a woman should discuss with a trusted elder or health professionals before making a decision (abortion).

Induced abortion could be risky and deadly. One should not trust illegal health providers and unlicensed doctors.

and admission of the obligation to raise awareness through communication including:

We need to have awareness on pregnancy prevention when having sexual relations.

Useful activities, should continue and expand more into other places or clusters, and to adolescents who are not able to attend school.

The experiences of adolescent pregnancy included comments on sexual relationships with some maintaining traditional attitudes:

To have self-restraint and self-discipline.

Girls should not trust boys easily on taking responsibility in relation to sex: the burden will be on a girl’s shoulders.

while others were more liberal:

Why didn’t he use a condom?

The feedback from community members to survey questions after drama performance was summarised (Table 2). The main messages aligned with the intended objectives of the drama and the plot was relatable. The drama was seen to offer a way for teenagers to ‘*consult with their parents*’ and for parents to ‘*help and discuss*’ how to ‘*solve problems*’ and where to ‘*get support and consultation*’. Overall, the drama was recommended for wider dissemination to ‘*show it to young people who do not go to school*’ and the method, with interactions based on dialogue with the audience, was considered a useful means to deliver ‘*any health-related topic*’ in the future. The Dream Scene was well received but the sensitive scene (shadow scene) raised the most negative responses e.g. ‘*inappropriate*’. These were countered by responses at the other end of the spectrum: ‘*no sensitive scenes’*. The sentiment of these statements is best captured in the following short video: https://youtu.be/-AGwHLYF0NQ.

**Table 2.** Feedback comments from the community post-drama.

Question	Responses/Comments
Feelings about this drama	“This is the first drama show ever conducted in the community and in the school.” “The drama is attractive and interesting and the story is practical.”
Main messages from the drama show	“The communities and adolescents gained sexual reproductive health knowledge and how to protect themselves.” “Providing sexual reproductive health knowledge as either training or curriculum in school to adolescents would be useful.” “The messages about the termination of pregnancy being legally approved by Thai Government is new information to migrants.” “If someone encountered the same issues or problems, they now know how to solve problems and where they can get further support and consultation.”
Relatability	“Parents do not take care of nor spend time with their children because they have to struggle with their daily work.” “Most adolescents in the community get married and pregnant early because of a lack of sexual reproductive health knowledge.”
Opinion about the dream scene;	“While experiencing an unwanted pregnancy. in the dream we see that it is better to first have a consultation with health professionals to make the right choice.” “That performance is very impressive.” “For some conservative or traditional communities, TOP [termination of pregnancy] is sensitive, it will probably encounter some negative comments.”
Opinion, Recommendation or Suggestion about sensitive scene	“I don’t think it’s suitable for young people especially the scene when they fall in love. I am worried that young people will take that as a normal example. I think it would be better to turn off the lights in that scene.” “Some scenes seem to show more than what is necessary. I don’t think it’s suitable for children. As for us adults, there is no problem.” “There are no scenes that are inappropriate. Today’s young people have more understanding. In a very conservative area, I think you can get negative views.” “No sensitive scenes. Those who come to see it with their own eyes make it more effective. It is effective because the show is straightforward.”
Knowledge and Understanding	“I am sure that my students will improve their knowledge of adolescent pregnancy. In the past, they talked about sexual reproductive health in school lectures, my students are tired of the lectures. Now they have seen the story with their own eyes and heard the questions asked in the drama show, it’s not easy to forget. They are interested.” “I’m sure they will have gained understanding and knowledge about adolescent pregnancy. After watching the drama, parents will be more careful about how to take care of children in the family. Young people look at themselves and they will be cautious and protect themselves.” “After watching, I gained knowledge about how we should teach our children and young people” “This drama enhances and increases the knowledge of sexual reproductive health in the community, students and adolescents.”
Creating a friendly environment to talk about adolescents	“I think that if there are problems like in the show, they will consult with their parents more openly, because these problems cannot be solved alone. It has to be solved by village leaders, parents, teachers, and health staff together.” “If there is a problem, how to solve it is already in the story, so instead of criticizing those around us, I think they will be open to helping and discussing.”
Health messaging	“I see that the plot makes it easy for everyone to understand. The migrant people are not very interested in what we explain to them. If you show this descriptive story, it reflects their life and so it will also be very effective. So, I think this can be effectively used for any health-related topic.”
Recommend this drama show to others and to use in the future	“Adolescents should see it; it is a must-see drama.” “It is better to show it also to young people who do not go to school.” “I want to show the parents, grandparents, and young adults who live in the village. They should also know the contents of this show. If they get the knowledge, they should be able to protect themselves or help the young people.” “Please continue conducting this impressive and interesting drama performance in many different places as it is not only similar to real situations but also opens the door for people who have been forced to cover up this issue in the past”. “To disseminate this drama performance more as the story has been seen in the time of our Grandparents Ages (it not a new story) but now they saw and heard the Drama Performance in person, and that really makes them remember”. “This critical and sensitive topic of sexual reproductive health *[adolescent pregnancy]* makes many troubles in the conservative Myanmar community. Not just communities only, but other organizations also have an obligation to transform by articulating this problem and introducing this change slowly for better results and visions”.

### Unexpected results or problems that arose from the presentation of the drama

The schedule of migrant daily work is unpredictable, so while the performance was arranged in advanced the audience was sometimes smaller than planned as work took priority for migrants. As there is no such thing as child care in the communities they were present at the drama when it was not appropriate for them (Table 2). Distractions for the audience and team affected concentration with some examples including: inappropriate cheering of some members of the audience and unwanted road noises due to the location of some migrant clusters. There were amplification issues at some of the performances. The team itself encountered: manpower shortages, particularly when there was a need to substitute actors who were sick with COVID-19; difficulty in sustaining the emotional energy required for the performance content. The need for an alternative to community drama performances was recognised as the physical demands and cost of moving the team out were burdensome for a team that has other work commitments.

### What the team learned from the process of developing and presenting the drama

The SMRU-BHF Drama team gained public performance confidence upon seeing the acceptability, excitement, and positive feedback after performing in Karen or Burmese language. The drama enhanced collaboration among the team members and partners at the migrant schools, the Young Person’s Advisory Group (Y-PAG) and its network which can be useful in the future as health system strengthening relies on partners. The team thinks the most likely reason for the positive response resulted from the PAR and ethnographic approach where community perceptions were probed initially and then used to develop the drama which made it respectful and relevant to the difficult life faced by migrants, while also showing the community through the dialogue that they know and need to be part of the solution.

## Discussion

The objectives of the drama to raise community awareness of adolescent pregnancy, contraception, and choice in the event of an unplanned pregnancy were met as reflected by the community members response to the drama. An encompassing response was: ‘*I’m sure they [students] will have gained understanding and knowledge about adolescent pregnancy. After watching the drama, parents will be more careful about how to take care of children in the family. Young people look at themselves and they will be cautious and protect themselves.’* The objectives of the drama were accomplished as supported by the positive perceptions of the drama among community members. For the objective of responsibility, mothers, parents, and teachers, in their relationship with adolescents, were all mentioned. For the objective of communication ‘ *… consult with their parents more openly …* ’, ‘ *… instead of criticizing … ’* emerged. For the objective of experience: ‘*If someone encountered the same issues or problems, they now know how to solve problems and where they can get further support and consultation*.’ This is a marked change from the experience of stigma voiced at baseline where: ‘*Adolescents felt there was gossip about them, they were scolded, criticized and experienced a sense of discrimination*.’

While the drama presented an opportunity to discuss adolescent pregnancy and making decisions about abortion, tradition, and taboo remained part of the comments after the drama. For example: ‘*To have self-restraint and self-discipline*’ and ‘*Girls should not trust boys easily on taking responsibility in relation to sex: the burden will be on a girl’s shoulders*’. These gender roles are identified here for the first time in this border area of south-east Asia and are consistent with the international literature in populations living in poverty [35], marginalised [36] and under restrictive abortion laws [37]. While the law has changed recently in Thailand it remains restrictive in Myanmar. The avoidance of responsibility by the lead male character Min Khant and the absence of comments on the role of fathers having a close relationship with their sons was consistent with cultural inequity observed in Thailand [38] and in Laos [39] where premarital sex for boys does not meet with the stigma that is applied to girls. This important social norm must be challenged in future engagement.

The drama for marginalised communities increased their reproductive health knowledge, informed them on how to prevent unwanted pregnancies, and provided information about safe abortion care. A windfall of the drama was a strong call by community members to show, teach, and disseminate the performance, as well as trainings and curriculum on sexual and reproductive health. This is best articulated in ‘*This critical and sensitive topic of sexual reproductive health [adolescent pregnancy] makes many troubles in the conservative Myanmar community. Not just communities only, but other organizations also have an obligation to transform by articulating this problem and introducing this change slowly for better results*’. This may be considered a step forwards from previous reports from the border where traditional views and stigma on SRH issues largely prevent discussion on these topics [11].

There were negative responses to the very brief shadow scene suggesting the young couple had a sexual relationship. While these negative responses were outweighed by positive responses, some community members deemed it unsuitable for young people as it may encourage them to be sexually active. Other respondents suggested that young people understand much more than what was shown, and one suggested that part of the effectiveness of the show was its ‘straightforward’ nature. While the drama team sought and expected feedback on the sensitive scene it brought one student to comment directly, ‘*why didn’t he use a condom*?’. The drama team could have made this a definitive question at dialogue time to highlight this critical moment of the drama because of the dire consequences for girls and their families, and the responsibility of the boy for his action.

The effect of COVID-19 on the informal migrant school sector was recognised in the fish tail as restriction and suppression of migrant activities and movements were severe, with two years of school closure [40]. The community itself stated ‘ … better to show it also to young people who do not go to school’ as some live in hard to reach areas and appealed for the drama to be made available to them [41]. It could be that the community recognises that adolescents who most need the messaging provided in the drama are not at school. Mechanisms to reach a broader target audience that would include those adolescents not attending school are under discussion with the community engagement team. Online video content has been suggested as a mechanism [42] although the team realises phone ownership and internet access are also limited for this population and some migrant clusters areas have no electricity supply at all. The drama team themselves recognized the difficulty of sustainability. Shifting to a video format is being considered for three reasons: the performance was emotionally draining for the actors; a more portable tool than an entire drama crew is needed to reach migrants in Tak Province including post-coup d’etat arrivals; and a tool that can cross borders will be more cost-effective and sustainable.

There were limitations to our study. The input from pregnant adolescents prior to the drama was not as high as hoped as COVID-19 restricted movement of the team to different migrant clusters for IDI and FGD. However, the input from adolescents after the drama was high. As youth were the target population, it is only through actively engaging youth in the process [43] that pertinent messaging can be developed. However, respecting traditional culture and promoting healthy adolescent-parent communications are also important to creating a healthier environment for this dialogue. We accept that some communities would not agree to having the drama, and that the non-explicit scene suggestive of sex was still ‘not suitable’ for some community members. However, these views still help the team to find the right pitch for this sensitive subject. While the show-of-hands method can be considered a limitation as it can be biased due to peer expectations, it was a non-threatening way to warm-up and encourage audiences to join in with the comments and feedback.

After understanding *a priori* the perceptions towards adolescent pregnancy in the community the highlight of this PAR was that the drama was positively received despite the inclusion of traditionally taboo and sensitive subjects such as adolescent pregnancy and safe abortion care [44]. On the border, the approach to this problem is innovative as the PAR which is also good practice, was conducted in Karen and Burmese language so cultural nuances of the original perceptions from community members pre drama fed into the fishbone [44], the narrative drama was developed locally [32,33], feedback after the performance was also expressed in local languages, with English being accessory and used to explain this process to a wider audience [45]. The success of the drama was likely due to the relatability of the fictional narrative to the community, which is previously recognised in the literature as essential for effective health messaging [4]. PAR with cross-sectoral cooperation and active community engagement has previously been promoted as a useful method to explore the causes of health issues [46] and determine solutions [47].

## Conclusion

This drama positively impacted the community by raising awareness of adolescent pregnancy, contraception, and choice in the event of an unplanned pregnancy. A PAR approach involving community members as well as adolescents resulted in a drama that was entertaining and stimulating for discussion of traditionally taboo issues like adolescent pregnancy and unsafe abortion. The local language and community driven dialogue-drama as an ethnographic method was recommended by the community themselves for other health issues. Sharing this process may be beneficial for other language diverse and resource-limited settings.

## Appendix Baseline perceptions interview - teen mother at antenatal care

1. Could you please introduce yourself?

*Probe:*

- May I know the name you would like us to call you in the interview? What is your age?

- Are you currently attending school? Why or Why not?

2. Is this your first ANC? Are you coming alone?

*Probe:*

- When did you know you were pregnant? How did you find out?

- Do you remember how you felt? Excited? Worried? Happy?

- Did you marry? Did you decide to marry? Did you consult anyone beforehand?

- Did you try any contraception? Why/Why not?

3. When did you get married? How did you meet your husband?

*Probe:*

- Could you please share with us about your life after getting married?

- Did you face any challenges/difficulties after getting married?

- What was your biggest challenge/difficulties/problems? How did you overcome that?

4. When you need help, where do you seek for support? (for example, information about sexual reproductive health, antenatal care, moral or social support …)

*Probe:*

- from the hospital or clinic, school, relatives, friends, or family etc.

- who do you usually consult or seek for support from? Why?

5. How do you see yourself as a (future) mother? What do you think other people think about you as a teen mom?

6. Have you observed many teen moms around you? If you could say something to adolescent women about pregnancy and married life, what will be your message/s? What would you tell them?

### Baseline perceptions interview/discussions - community members with experience of adolescent pregnancy

1. Do you observe many adolescent pregnant women in your community?

2. What do you think about it?

*Probe:*

- Why do you think it happens?

- Do you see it as normal, as a problem?

- Do you think we should do anything about it?

3. Have you ever been asked by a teenager (your son, daughter, niece, nephew, neighbour etc …) about love, sex, romantic relationship, marriage etc? or any problem related to these?

4. What do you think when young people come and consult you on these topics?

*Probe:*

- How do you feel? Do you feel comfortable answering the questions? Why or why not?

- What was your suggestion/ideas/support … that you gave to them.

5. Do you want to say something about teenage pregnancy?

*Probe:*

- What ‘changes’ do you want to see regarding teenage pregnancy?

### Baseline perceptions discussion - SMRU-BHF health care staff providing services for adolescent pregnancy

1. What do you think about adolescent pregnant women coming to the clinic?

2. Could you please share with us your experience giving a service to adolescent pregnant women?

*Probe:*

- Any challenges before, during and after of giving the service?

- Can you tell me the challenges that adolescent women faced when they come to the clinic?

3. Have you ever been asked by a teenager (your son, daughter, niece, nephew, neighbour etc …) about love, sex, romantic relationship, marriage etc? or any problem related to these? What do you think when young people come and consult you on these topics?

*Probe:*

- How do you feel? Do you feel comfortable answering the questions? Why or why not?

- What was your suggestion/ideas/support … that you gave to them.

4. Do you think our clinics provide enough support to a teen mom/pregnant woman?

*Probe:*

- Do you think that our clinic is a friendly place where a teen mom/pregnant woman feel comfortable to discuss their problems related to teenage pregnancy (sex, relationship, fear, anxiety, family planning, contraception, information etc)

- Is there anything to be improved?

5. If you could say something to adolescent women about pregnancy and married life, what will be your message/s? (or what will you tell them?)
